# Diabetes-induced oxidative stress in the vitreous humor

**DOI:** 10.1016/j.redox.2016.07.003

**Published:** 2016-07-08

**Authors:** Zsuzsanna Géhl, Edina Bakondi, Miklós D. Resch, Csaba Hegedűs, Katalin Kovács, Petra Lakatos, Antal Szabó, Zoltán Nagy, László Virág

**Affiliations:** a Department of Ophthalmology, Semmelweis University, Budapest, Hungary; b Department of Medical Chemistry, Faculty of Medicine, University of Debrecen, Debrecen, Hungary; c MTA-DE Cell Biology and Signaling Research Group of the Hungarian Academy of Sciences, Debrecen, Hungary

**Keywords:** Diabetes, Vitreous, Protein carbonylation, Protein oxidation, Advanced glycation endproducts, Antioxidants, Glutathion

## Abstract

**Purpose:**

Diabetes is accompanied by fundamental rearrangements in redox homeostasis. Hyperglycemia triggers the production of reactive oxygen and nitrogen species which contributes to tissue damage in various target organs. Proliferative diabetic retinopathy (PDR) is a common manifestation of diabetic complications but information on the possible role of reactive intermediates in this condition with special regard to the involvement of the vitreous in PDR-associated redox alterations is scarce.

The aim of the study was to determine key parameters of redox homeostasis [advanced glycation endproducts (AGE); protein carbonyl and glutathione (GSH)] content in the vitreous in PDR patients.

**Methods:**

The study population involved 10 diabetic patients undergoing surgery for complications of proliferative diabetic retinopathy and 8 control (non-diabetic) patients who were undergoing surgery for epiretinal membranes. Vitreal fluids were assayed for the above biochemical parameters.

**Results:**

We found elevated levels of AGE in the vitreous of PDR patients (812.10 vs 491.69 ng AGE/mg protein). Extent of protein carbonylation was also higher in the samples of diabetic patients (2.08 vs 0.67 A/100 μg protein). The GSH content also increased in the vitreous of PDR patients as compared to the control group (4.54 vs 2.35 μmol/μg protein), respectively.

**Conclusion:**

The study demonstrates that diabetes-associated redox alterations also reach the vitreous with the most prominent changes being increased protein carbonylation and increased antioxidant levels.

## Highlights

• Vitreal AGE levels are elevated in PDR patients.
• Extent of protein carbonylation is higher in the vitreal samples of PDR patients.
• The glutathione content is increased in the vitreous of PDR patients.

## Graphical abstract

**Figure:**
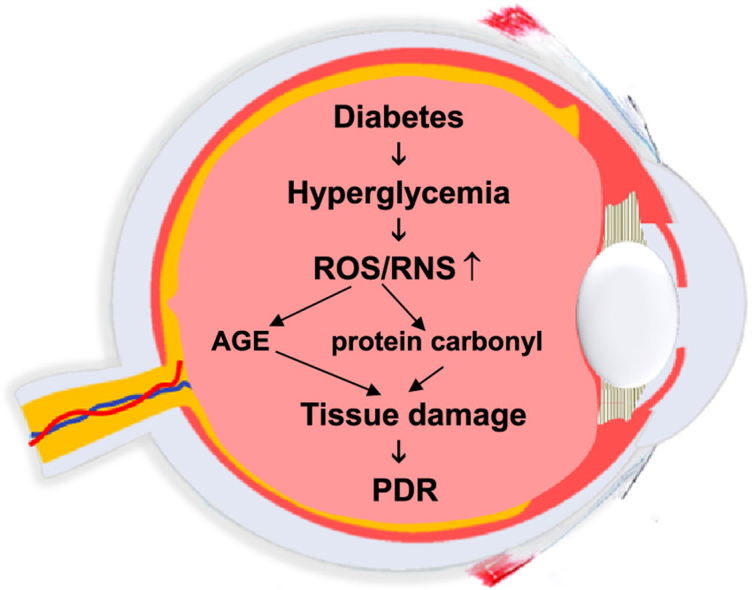

## 1 Introduction

Diabetes is one of the most prominent diseases in the world and in recent decades its incidence reached “epidemic” proportions. According to WHO the global prevalence of diabetes is 9% of adults aged 18 years or more [1]. In contrary to common belief the diabetes-related medical and health care problems and the associated societal challenges are not restricted to wealthy societies as indicated by the fact that 80% of diabetes deaths occur in middle or low-income countries [2].

While the efficient treatment of the disease itself especially that of insulin resistance in type 2 diabetes cannot be considered as a solved medical problem, it is the management of diabetic complications (e.g. vasculopathy, neuropathy, nephropathy) that truly represents an unmet medical need. The eye is an important target organ often affected by diabetic complications with diabetic retinopathy being the leading cause of blindness in the developed countries [3]. In 5–10% of diabetes patients proliferative diabetic retinopathy (PDR) develops [4] affecting mostly type I diabetics. PDR is one of the most severe diabetic complications [5] characterized by abnormally proliferating retinal blood vessels, neovascularization and cell proliferation [6]. It often leads to vitreal bleedings and tractional retinal detachment culminating in severe deterioration of vision [7].

Several mechanisms [e.g. genetic factors, increased vascular endothelial growth factor (VEGF) production, altered extracellular matrix architecture, redox signaling] have been demonstrated to underlie angiogenesis, collateral vessel formation and increased permeability observed in PDR [8], [9], [10]. Production of various reactive oxygen and nitrogen species (ROS and RNS, respectively) have been shown to occur in diabetic tissues including the retina [11], [12]. ROS have also been implicated in diabetes-associated angiogenesis in the retina and are considered important contributors to diabetic retinopathy [13], [14].

In our present study we set out to characterize the redox environment in the vitreous humor of PDR patients. As indicator of glycemic control we measured AGE, as a sign of oxidative damage we determined protein oxidation, whereas the antioxidant status has been assessed by measuring glutathione levels.

## 2 Materials and methods

### 2.1 Patient groups and tissue samples

All procedures performed as part of this study were in accordance with the ethical standards of Semmelweis University and with the 1964 Helsinki declaration and its later amendments or comparable ethical standards. The study has been approved by ETT-TUKEB (Scientific and Research Committee of the Medical Research Council, Hungary) under protocol number: 9683-1/2012/EKU. Informed consent was obtained from all individual participants included in the study.

The study population involved diabetic patients (n=10; 6 males and 4 females) with an average age of 54 (range 34–69) and the control group (n=8; 2 males and 6 females) operated with epiretinal membranes. Average age of control patients was 72 (range 67–80). In the diabetes group (7 type 2 diabetic patients and 3 type 1 diabetic patients with all but one patient under insulin treatment) the disease has persisted for an average of 17.7 years (2–34) and surgery was performed due to complications of proliferative diabetic retinopathy (bleeding, retinal ablation, proliferation bundles). With regard to the higher average age of control patients it is important to note that according to our current understanding, the age of the diabetic patients does not significantly affect the phenotype of diabetic retinopathy [15]. Moreover, it has also been reported that total protein carbonyl levels don’t correlate with age [16] and the ophthalmological features of diabetic retinopathy are independent from the type of diabetes [17].

Undiluted vitreal samples were obtained by pars plana vitrectomy and samples were stored in eppendorf tubes at −80 °C until use.

### 2.2 Determination of Advanced Glycation End Products (AGE)

Amount of glycated proteins was determined using the OxiSelect Advanced Glycation End Product Competitive ELISA Kit (Cell Biolabs, Inc., San Diego) following the manufacturer's instructions.

### 2.3 Protein carbonylation

ROS and RNS can modify amino acid side chains of proteins to carbonyl (aldehyde or ketone). For protein carbonyl determinations, each sample was diluted with distilled water to reach a concentration of 20 μg protein in 200 μl. Then 50 μl of 80% trichloroacetic acid (TCA) solution was added, samples were vortexed and incubated on ice for 5 min. After centrifugation at 13,000×*g* for 2 min, supernatants were removed and pellets were resuspended in 500 μl ice-cold acetone. Incubation for 5 min at −20 °C was followed by another centrifugation at 13,000×*g* for 2 min. Acetone was removed and pellets were dissolved in 20 μl distilled water and used for protein carbonyl detection using OxyBlot Protein Oxidation Kit (Merck Millipore, Budapest, Hungary) following the manufacturer's instructions. After completion of the derivatization step, the obtained dinitrophenol (DNP) product was quantified spectrophotometrically by measuring optical density (OD) at 405 nm.

### 2.4 Glutathion assay

Samples were precipitated for 10 min with ice cold 10% TCA solution. After centrifugation (5000 g, 4 °C) supernatant was used for further analysis. The glutathione (GSH) assay was performed in 96-well plates. Potassium phosphate buffer (1 M) and o-phthalaldehyde (0.5%) were added to the samples and after 30 min incubation at room temperature fluorescence was measured at 390/460 nm. The mixture of supernatant and N-ethylmaleimide was used as a blank for each sample. Standard curve was prepared with GSH. Protein concentration was determined with the BCA (bicinchoninic assay) method.

### 2.5 Statistical analysis

Concentration values of vitreous samples obtained from diabetic and non-diabetic patients were compared by Statistica 11.0 (Statsoft, Tulsa OK, USA) software using the Mann-Whitney *U* Tests. Difference was considered significant if p<0.05.

## 3 Results

### 3.1 Glycation products in the vitreous

**Fig. 1:**
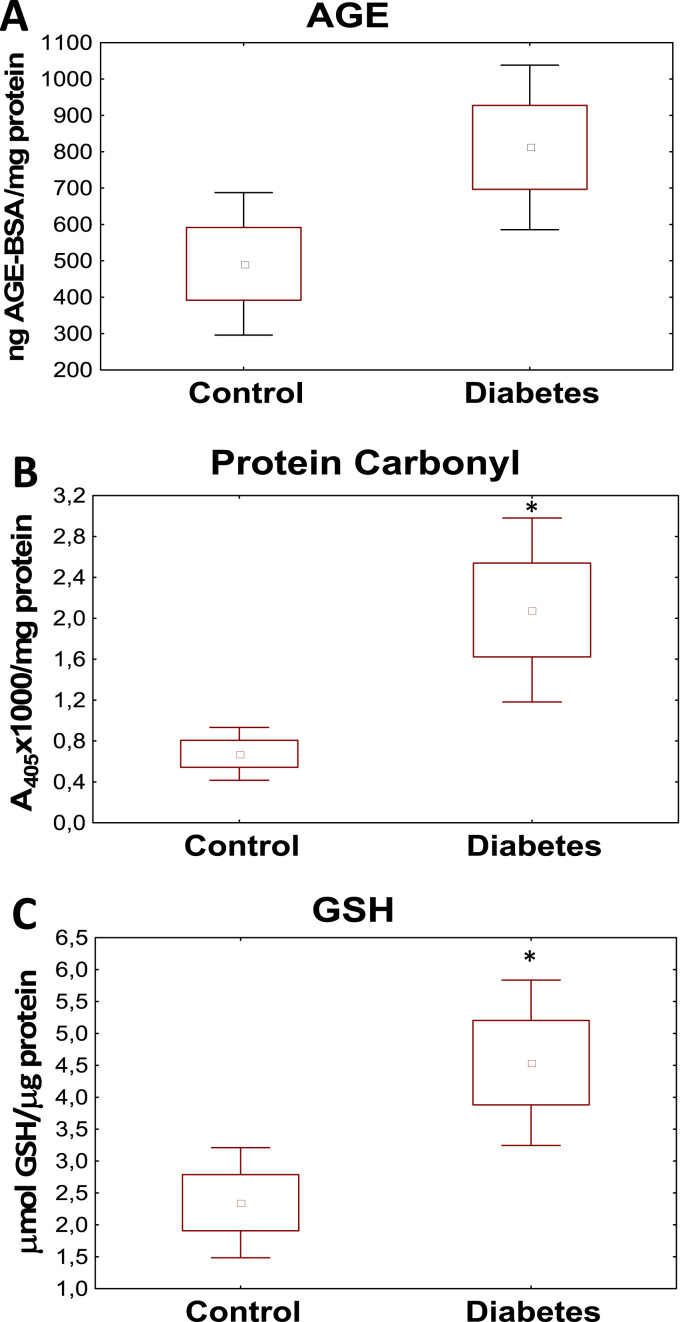
Vitreal samples were collected from diabetic and control patients and advanced glycation endproducts (AGE) (A), protein carbonylation (B) and reduced glutathione (GSH) content (C) have been determined as described in the Methods section. Star signs indicate significant difference between groups (^⁎^p<0.05, Mann-Whitney *U*-Test).

Efficiency of glycemic control in diabetics is mirrored by serum (and tissue) levels of AGEs [18]. AGEs are stable endproducts formed in a non-enzyme catalyzed reaction between reducing sugars such as glucose and amino groups of proteins (or lipids). The Schiff base formed in this reaction may in turn undergo further chemical rearrangements to form stable Amadori products. Glycated proteins (e.g. glycated hemoglobin A1c) are extensively used in the clinical praxis to provide information about serum glucose levels of the past 6–8 weeks [19]. Since to our best knowledge AGE levels have not yet been measured in the vitreous, we set out to investigate whether increased glycation can also be detected in the vitreous of PDR patients. We found increased AGE levels in the vitreous of PDR patients (Fig. 1A) compared to control samples (obtained from patients undergoing surgery for epiretinal membranes) 812.10 vs 491.69 ng/mg protein (p=0.058). This finding indicates that glucose levels in the vitreous display similar changes to that in the serum and other body compartments.

### 3.2 Oxidative stress in the vitreous

Oxidative stress is often defined as an imbalance between the production and elimination of ROS and RNS. If the antioxidant capacity of a tissue is not sufficient to prevent ROS/RNS hitting biological targets then various oxidative and nitrative protein, lipid and DNA modifications may occur [20], [21], [22], [23], [24]. Appearance of carbonyl groups on amino acid side chains (typically those of proline, lysine, arginine and threonine) is regarded as a sign of oxidative protein damage that can be triggered by a variety of ROS and RNS species [25]. Protein carbonyl formation has been shown to be an important marker in various oxidative stress paradigms [25] and to contribute to cell dysfunction in various diseases including different types of diabetic cellular dysfunction [26], [27].

Here we have determined protein carbonyl content of vitreous samples (Fig. 1B). We found that protein carbonylation is significantly increased in the samples of diabetic patients as compared to control (2.08 vs 0.67 A_405_/100 μg protein; p=0.017). This finding indicates that oxidative stress is present in the vitreous of diabetic patients and ROS/RNS species can react with proteins in this compartment.

### 3.3 Antioxidants in the vitreous

In addition to the production of ROS and RNS the actual condition and composition of the antioxidant repertoire is also a key determinant of the redox state of a compartment. Therefore we also set out to obtain information on how well the vitreous of diabetics is equipped with antioxidants. We have determined the concentrations of the major endogenous antioxidant glutathione in the vitreal samples (Fig. 1C). We found that the levels of reduced glutathione (GSH) were also significantly (p<0.05) higher in diabetic vitreous as compared to control (4.53 vs 2.34 μmol/μg protein; p=0.019).

## 4 Discussion

Even in case of close-to-ideal diabetes management, serum glucose levels cannot always be kept at optimal levels. Temporary glycemic swings may be sufficient to increase protein glycation in the blood and in various tissues [28]. Several lines of evidence indicate that in diabetes AGEs form in the eye and contribute to diabetic complications [29]. This is most prominent in the retina where antioxidant enzymes, transcription factors and mitochondrial proteins have been found to undergo glycation which may impair their functions [30], [31]. Thus protein glycation may contribute to oxidative stress in the diabetic eye [32]. Our finding that AGE levels increase in the vitreous suggests that glucose levels are increased in this ocular compartment. Since metabolic exchange and equilibration between systemic circulation and vitreous humor is considered to be slow [33], elevated vitreal AGE levels may indicate occurrence of sustained hyperglycemic periods in our study patients. By analogy to what we know about the mechanism of oxidative stress in the retina [34], it may be plausible to hypothesize that glycation of vitreal proteins may also contribute to the development of vitreal oxidative stress.

Tissue oxidative stress is typically accompanied by oxidative protein, DNA and lipid modifications [35]. We measured protein carbonyl content as protein oxidation marker. Elevated protein carbonyl levels in the vitreous of diabetic patients provide further support for the hypothesis that the vitreous humor is no exemption from oxidative diabetic environment in the eye. Vitreous humor abounds in a high variety of proteins with albumin and type II collagen being the most notable ones [36]. Thus ROS/RNS may hit protein targets with high probability.

In oxidative stress situations antioxidants are often depleted by continuous attack of prooxidant stimuli [37]. Therefore we expected lower antioxidant activities in the vitreous of diabetic patients. In the contrary, we found that vitreal level of reduced glutathione was increased in the diabetic patients. This may indicate an adaptive response to increased ROS/RNS production just like it has been reported for the extracellular antioxidant enzyme superoxide dismutase in PDR [38]. Glutamate cysteine ligase, the key enzyme of glutathione synthesis is under control of Nrf2, the master regulator of redox homeostasis [39]. Therefore, sustained, chronic oxidative stress may in the long-term induce an Nrf2-mediated upregulation of GSH synthesis. To find out whether or not this is the case requires further investigation.

Overall our study highlights significant rearrangements in the redox homeostasis of human vitreal fluid of diabetic patients with possible implications for the pathomechanism of PDR.

## Authors' contributions

Zsuzsanna Géhl designed the study, collected samples, evaluated data; has written the draft of the manuscript.

Edina Bakondi coordinated labwork, performed experiments, evaluated data.

Miklós Resch collected samples, performed statistical evaluation of data, critically evaluated data.

Csaba Hegedűs performed experiments, has written the manuscript.

Katalin Kovács performed experiments.

Petra Lakatos performed experiments.

Antal Szabó collected samples, critically evaluated data.

Zoltán Nagy contributed to study design, critically evaluated data.

László Virág designed the study, evaluated data, has written the draft of the manuscript.

All authors participated in drafting the article and critically revising it.
